# Kikuchi Fujimoto Disease, a Worrisome Presentation With a Reassuring Outcome

**DOI:** 10.4021/wjon292e

**Published:** 2011-04-09

**Authors:** Zaher Oueida, Ioana Chirca, David Gallinson

**Affiliations:** a Department of Internal Medicine, Morristown Memorial Hospital, 100 Madison Ave, Morristown, NJ 07962, USA; b Department of Hematology/Oncology, Morristown Memorial Hospital, 100 Madison Ave, Carol Cancer Center, Morristown, NJ 07962, USA

**Keywords:** Kikuchi, Fujimoto, Histiocytic, Lymphadenitis

## Abstract

We describe the case of a 27-year-old male with painful lymphadenopathy involving multiple sites. An excisional lymph node biopsy established the diagnosis of Kikuchi-Fujimoto disease (KFD) and the patient improved with supportive care only and did not have further episodes. This is a case of a rare, benign lymphadenitis, of unknown etiology. The histopathology proved the benign character of the lymphadenopathy and made the diagnosis clear, despite the nonspecific character of the clinical presentation. It is important to recognize the clinical and histopathological aspects of KFD in order to avoid unnecessary and sometimes harmful treatment, such as chemotherapy, when this disease is confounded with lymphoma.

## Introduction

Histiocytic necrotizing lymphadenitis or Kikuchi-Fujimoto disease (KFD) [1, 2] is an idiopathic and benign lymphadenitis classified as one of the atypical lymphoid hyperplasia mimicking lymphoma [3].

The etiology and pathophysiology of the disease are so far unknown but infectious as well as autoimmune mechanisms were speculated [4-7]; it was even proposed as self-limited systemic lupus-like autoimmune condition caused by virus-infected transformed lymphocytes [8]. The vast majority of literature consists of case reports and case series as this is a rare condition.

KFD disease almost always runs a benign course and resolves in several weeks to months.

## Case Report

This is the case of a 27-year-old Hispanic man with no significant past medical history who presented with five days history of painful laterocervical adenopathy, accompanied by fever, chills, night sweats and rhinorrhea. Review of systems was negative for cough and weight loss. He has no sick contacts, recent travel or occupational exposure. He is a non-smoker, non-drinker and his sexual activity is monogamous.

On admission he was afebrile and in no acute distress. He had a macular, erythematous rash with some squames on bilateral shoulders and anterior chest. No pharyngeal erythema, enlarged tonsils or oral thrush were noted. Papilledema and nuchal rigidity were also absent on exam. He had bilateral cervical and submandibular lymphadenopathy, left more than right. There was a two centimeters ganglionar block in the posterior laterocervical region, rounded in shape, rubbery in consistency, non-erythematous, non-adherent to the sub- and suprajacent structures and tender to palpation. There were also left axillary and bilateral inguinal mildly enlarged lymph nodes. No hepatosplenomegaly was appreciated and the rest of the physical exam was unremarkable.

His laboratory work showed borderline decreased white count (4,500/dl) with 14% mononuclears, 28% lymphocytes and 46% neutrophils and mildly elevated lactate dehydrogenase. Rapid strep test was negative and Chest X-ray was unremarkable.

He was admitted to the hospital and the differential diagnosis included infectious, malignant and rheumatologic etiologies. The HIV test was negative, as well as Monospot, rapid plasma reagin, anti-nuclear antibodies, and rheumatoid factor.

A computed tomography (CT) scan of thorax, abdomen and pelvis confirmed the sole involvement of the sites previously described in the physical exam.

An excisional lymph node biopsy showed paracortical hyperplasia with multiple confluent areas of patchy necrosis, containing eosinophilic cellular debris, nuclear dust and a large number of pale-staining histiocytes admixed with small lymphocytes. Immunostaining for CD68 confirmed the presence of numerous histiocytes. There was a predominance of suppressor cells (CD8) over helper cells (CD4). All these findings were diagnostic of histiocytic necrotizing lymphadenitis.

The patient clinically improved with supportive treatment and he was discharged with primary care follow-up. He had no further episodes to date.

## Discussion

It is worrisome for a young individual to present to medical attention with diffuse lymphadenopathy of insidious onset, suggesting an oncologic or infectious etiology. They were both ruled out by tissue biopsy consistent with KFD.

Even though the disease is usually described in Asian and Caucasian population [9], this patient was of Hispanic origin.

Clinically, the patient had lymphadenopathy involving multiple sites (bilateral cervical, submandibular, axillary, and bilateral inguinal) which may suggest hematologic malignancy and is very unusual for KFD. In a series of 108 patients, 83 had lymphadenopathy localized to one site, usually cervical and particularly the posterior cervical chain; only three patients had bilateral cervical adenopathy [9]. The lymph nodes (LNs) are usually 1 to 2 cm in diameter but occasionally are much larger (≤ 7 cm) [10]. The nodal enlargement is often associated with dull or acute pain, as was the case here.

The macular, erythematous, desquamative rash, even though nonspecific, was described in about one third of KFD patients and resolved without intervention, same as in this patient [11-13].

Many infectious etiologies were thought to be associated to KFD. Examples are Epstein Barr Virus [4, 5], Human Herpes Virus 6 and 8 [6], Human Immunodeficiency Virus (HIV), Parvovirus B19 [7], Paramyxoviruses, Parainfluenza virus, Yersinia enterocolitica and Toxoplasma gondii. In the same time, HIV infection and infectious mononucleosis themselves can present in the same fashion but they were both tested negative. The clinical picture was not suggestive of any of the other etiologies.

Leukopenia is not very common in KFD (20 to 32%) [14]. Atypical lymphocytes on peripheral smear were reported in up to 25% of KFD patients [10]. A mildly elevated level of lactate dehydrogenase, even though non-specific was also noted [15].

On CT scan, this patient had perinodal infiltration, as well as areas of focal necrosis, rarely found in KFD patients. In one study, CT scan findings on 96 patients with KFD were: homogeneous lymph node enlargement in 83.3% of the patients, perinodal infiltration in 81.3%, and prominent areas of focal necrosis in 16.7% [16].

The patient underwent an excisional LN biopsy which revealed the diagnosis by showing the typical histopathologic findings in KFD. Tissue diagnosis is recommended by excisional biopsy instead of fine needle aspiration (FNA), since the latter has most often nonspecific findings and the overall accuracy in diagnosing Kikuchi disease is 56.75% [17].

Three phases were described in the literature by histology over the course of the disease: the proliferative phase (follicular hyperplasia with paracortical expansion), the necrotizing phase (paracortical necrosis, karyorrhexis and hystiocytic predominance) and the xanthomatous phase (resolution of necrosis). Immunohistochemical stains show CD68 positive plasmacytoid monocytes and histiocytes with predominantly CD8 positive T lymphocytes [18]. This case fits into the necrotizing phase, and the large prevalence of histiocytes was confirmed by immunohistochemical staining showing CD68 Positive cells.

This case brings into attention the importance of an accurate histopathologic diagnosis in a patient with lymphadenopathy of uncertain etiology. A clinical presentation as above is always worrisome, a hematologic malignancy or disseminated tuberculosis being always on top of the differential diagnosis. In this case the histopathology proved the benign character of the lymphadenopathy and made the diagnosis clear, despite the nonspecific character of the clinical presentation. It is important to recognize the clinical and histopathological aspects of KFD in order to avoid unnecessary and sometimes harmful treatment.
